# The care center influences the management of lymphoma patients in a universal health care system: an observational cohort study

**DOI:** 10.1186/s12913-016-1553-9

**Published:** 2016-08-02

**Authors:** S. Lamy, C. Bettiol, P. Grosclaude, G. Compaci, G Albertus, C. Récher, J. C. Nogaro, F. Despas, G. Laurent, C. Delpierre

**Affiliations:** 1 University of Toulouse III Paul Sabatier, Toulouse, France; 2 Department of Clinical Pharmacology, Toulouse University Hospital, Toulouse, France; 3 INSERM UMR1027 (The French National Institute of Health and Medical Research), Toulouse, France; 4 Department of Hematology, Toulouse University Hospital, Toulouse University Cancer Institute (IUCT-O), Toulouse, France; 5 Tarn Cancers Registry, Albi, France; 6 INSERM UMR1037 (The French National Institute of Health and Medical Research), Cancer Research Center of Toulouse, Toulouse, France

**Keywords:** Lymphoma, Center-related management disparity, Relative dose intensity, Observational cohort, French current practice

## Abstract

**Background:**

Healthcare providers-related disparities in adherence to the treatment plan among lymphoma patients are found even in a universal healthcare system, but the mechanism remains unclear. We investigated the association between the type of care center and the relative dose intensity and determined whether it persists after adjustment for patients’ recruitment differences.

**Methods:**

Prospective observational cohort study of 294 patients treated with standard protocols for diffuse large B-cell lymphoma (DLBCL) in teaching or community public hospitals or in private centers in the French Midi-Pyrénées region from 2006–2013. To test our assumptions, we used multinomial and mixed-effect logistic models progressively adjusted for patients’ biomedical characteristics, socio-spatial characteristics and treatment-related toxicity events.

**Results:**

Patients treated using standard protocols in the teaching hospital had more advanced stage and poorer initial prognosis without limitation regarding the distance from the residence to the care center. Patients’ recruitment profile across the different types of care center failed to explain the difference in relative dose intensity. Low relative dose intensity was less often observed in teaching hospital than elsewhere.

**Conclusion:**

We showed that even in a universal healthcare system, disparities in the management of DLBCL patients’ do exist according to the types of care center. A main issue may be to find and diffuse the reasons of this benefit in cancer management in the teaching hospital to the other centers.

## Key message

In this study in French current practice on 294 lymphoma patients treated with the standard R-CHOP or R-CHVP protocols, low relative dose intensity was less often observed in teaching hospital than elsewhere. This suggests that even in a universal healthcare system, disparities in the management of diffuse large B-cell lymphoma patients’ do exist according to the types of care center.

## Background

Little is known about the mechanisms behind survival disparities according to many non-medical factors including age, ethnic group, socioeconomic status (SES), and place of residence observed in hematological malignancies. [1–10] Among lymphoma, one potential mechanism emerges from the results of studies among Hodgkin lymphoma (HL) [11] and non-Hodgkin Lymphoma (NHL) [12–14] showing that socioeconomic factors, place of residence, and distance to the care center may affect access to treatment and treatment selection which was in turn related to survival. Another potential mechanism emerges from the results of studies dealing with the type of care provider and its consequence on survival. A population-based study of the Nebraska Lymphoma Study Group found a higher risk of death among high-risk lymphoma patients treated in rural university or community-based providers and urban community based provider compared to urban university-based providers. [15] More recently, in a study based on an ongoing French regional cohort of diffuse large B cell lymphomas (DLBCL) treated with R-CHOP (rituximab, cyclophosphamide, doxorubicin, vincristine, prednisone) or RCHOP-derived regimens, the results showed that care facility type may influence overall survival and patients management. In this study, patients cared for in the regional public teaching hospital were more likely to receive the dose of chemotherapy initially planned by the medical team, i.e. the relative dose intensity (RDI). [16] The ability to ensure a high RDI may be seen as a marker of high quality of care, as it is known to be associated with higher survival. [17, 18] To go further in the mechanisms linking the healthcare providers to survival, it remains uncertain whether better high RDI is only linked to a difference in patients’ recruitment profiles across the various types of treatment provider or whether it also reflects real differences in cancer care and management practices. Indeed, in France the secondary care in oncology consists of private funding centers which can be nonprofit or for-profit and nonprofit public funding centers encompassing small community public hospitals to larger university affiliated hospitals. Moreover, in the French healthcare system, based on a universal access to care, patients are able to choose freely the hospital they wish to go to. Thus, although all patients may have access to specialized care, some patients could miss out on the best possible treatment because of high preference for proximity centers. [19] This, with the fact that the academic or high-volume center are unlikely to be located outside large urban areas, may also lead to differences in the recruitment profiles of patients in the different types of care facility.

In the present study, we rule out both access to treatment and treatment selection issues by considering a sample of DLBCL patients all treated by standard protocols R-CHOP or R-CHVP (rituximab, cyclophosphamide, doxorubicin, etoposide, prednisone) each 21 days which constitutes a sample of patients globally homogeneous regarding the treatment their received [20, 21]. We aim to investigate whether and why RDI varies according to the types of care facility wherein patients were treated. Using data from French common practice, we try to disentangle the expression of patients’ recruitment disparities between centers from the expression of differences in centers’ features, which are assumed to be quite homogeneous for each type of care facility.

## Method

### Study design and population

The data were taken from the AMARE cohort, an ongoing prospective cohort of DLBCL patients in the Midi-Pyrénées region, the largest region of mainland France. This regional cancer network made up exclusively of cancer patients, including hematological malignancies, dictates that all e-medical files are systemically screened by disease-specific boards of experts during a multidisciplinary team meeting (MTM). Patients were identified by their presentation in MTM and were included in the AMARE cohort if they received first-line treatment for DLBCL with R-CHOP or R-CHOP-like regimens from November 2006 without age restriction, in the main centers covered by the regional cancer network: the public funding regional teaching hospital, five public funding community centers, and five private funding for-profit centers. The exclusion criteria were: central nervous system involvement, HIV infection, solid organ transplantation or previously documented indolent NHL. All patients signed an informed consent before inclusion in this network. The study was approved by the local ethics committee of the Toulouse teaching hospital. In the present study, we focused exclusively on patients treated by R-CHOP or R-CHVP which represented one on two patients treated for DLBCL of the initial AMARE cohort per types of care facility.

### Data collection

Data were collected by one person through direct examination of administrative and medical records of the 336 patients treated from November 2006–November 2013 (last follow-up in September 2014) who received the standard R-CHOP or R-CHVP protocols. During the follow-up, information was gathered regarding treatment-related events and vital status, including the date of the events. Care centers were pooled by type of care center: the public funding teaching hospital (Toulouse public funding University Hospital (UH)), the public funding community hospitals (CH), and the private funding for-profit hospitals (PH). Although these study deals with the data from 11 hospitals, we assume that the inter-facility type variability of the centers features are greater than the intra-facility type variability.

We collected patients’ biomedical characteristics at diagnosis: age (continuous and in quartile); comorbidity (none or at least one among chronic or viral hepatitis, cardiovascular or metabolic disease, autoimmune disease or cancer); gender; the Ann Arbor stage, divided into localized (Ann Arbor stage I or II) or advanced (Ann Arbor stage III or IV); the ECOG performance status (PS) (PS of 0–4, with 1 being good and 4 being poor) [22] and the serum lactate dehydrogenase concentration (normal or elevated (>1)). We preferred using direct values of these parameters rather than crude categorization as IPI [23, 24] or NCCN-IPI [25] scores that may introduce undesirable colinearity. We also noted the chemotherapy regimen at treatment initiation, which was described in detail elsewhere. [21, 26–28] Regarding patients’ socio-spatial characteristics, we used quintiles of the European ecological deprivation index (EDI) aggregated at the intra-municipalities level, built from patients’ residential addresses at diagnosis as a proxy of their individual socioeconomic status. [29] We assessed the travel distance by the road based on the fastest route in kilometers between the patient’s residence and their care center and between the patient’s residence and the teaching hospital coded in tertile to avoid making assumptions on any threshold. For each patient, the relative dose intensity (RDI) as described by Epelbaum et al. [30], was assessed using the ratio between the dose intensity received and that initially planned at the treatment initiation. In this study, we calculated RDI for the principal drugs, i.e. cyclophosphamide and doxorubicin. After excluding patients who deceased or had tumor progression during the first-line treatment, patients who did not receive the whole initially planned dose intensity (RDI < 100 %) were considered as having low RDI. Sensitivity analyzes were done using the continuous RDI value. Data regarding treatment-related toxicity events were documented by the total number of hospitalization days for toxicity over the first-line treatment period, but the dates of events were not available. The information regarding the cause concession was not available.

### Statistical analysis

To study the factors influencing patients’ repartition across the different types of care center, we built a multivariate multinomial logistic regression model by fixing the UH as the reference center to analyze the factors associated with being cared for in CH (model 1a) and in PH (model 1b). Then, we used multivariate logistic regression models with mixed effects to test for any center effect using the center as a random intercept. We tested the influence of the types of care providers on the probability of having low RDI without adjustment (model 2a) and after progressively adjusting for patients’ biomedical characteristics (model 2b), socio-spatial characteristics (model 2c) and treatment-related toxicity events (model 2d). Our models included all variables associated with these outcomes in bivariate analyzes at the threshold of 0.2 (data not shown). The models were systematically adjusted for age, sex and socioeconomic status. All analyzes were done by using STATA release 12 (StataCorp LP, College Station, TX, USA). The cartography of patients and center distribution was performed using R [31] and data from the French National Institute of Geography.

## Results

**Fig. 1:**
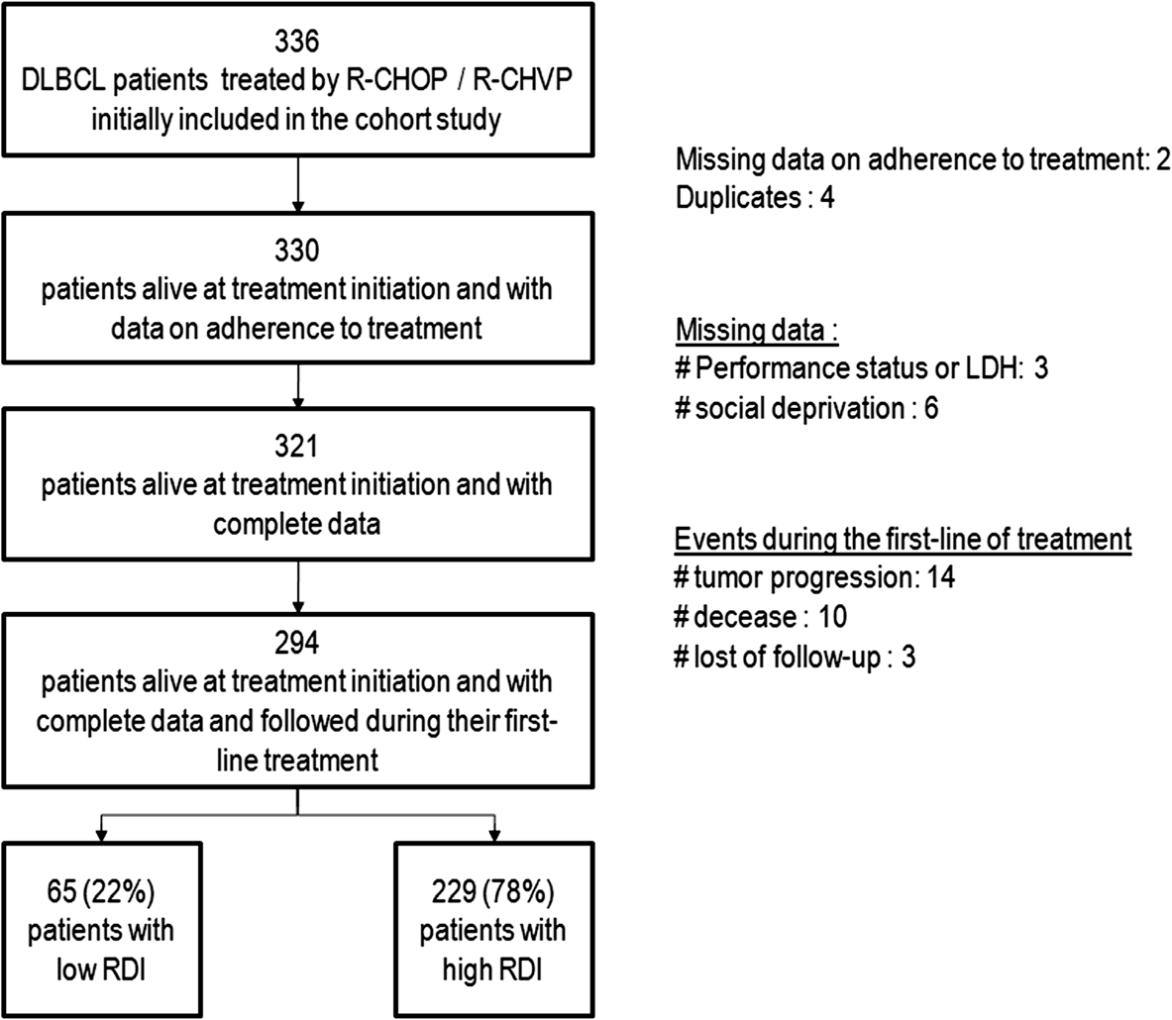
Flowchart

**Fig. 2:**
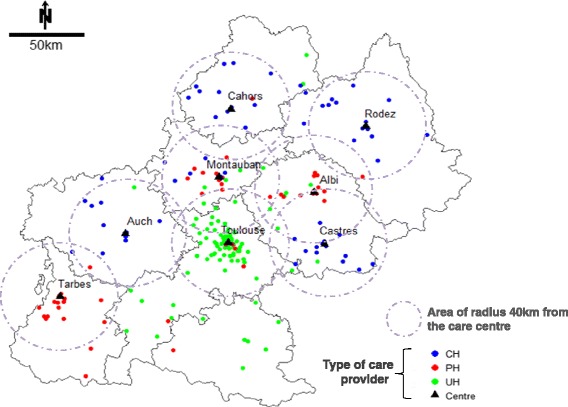
Patients overall spatial distribution by care providers

In this study, we analyzed 294 patients who were alive at the treatment initiation and had complete data, which was about 87 % of the total number of the 336 patients treated by R-CHOP or R-CHVP. Compared to the included patients, those who were not included in the study belonged more often to the oldest age class, had more often advanced stage tumor, had poorest initial prognoses, and presented more often hospitalization for treatment-related toxicity and poor RDI. However they were evenly spread through the types of care facility (data not shown). The flowchart is given in Fig. 1. The characteristics of patients, disease and cancer management by types of care center are presented in Table 1. Overall, the UH cared for younger patients with poorer initial prognosis, PH cared for patients with less comorbidity and better performance status, and CH cared for patients with more comorbidities and poorer performance status. Lastly, CH and PH tended to care for patients living in their vicinity, three out of four patients lived within a roughly 40 km radius, whereas patients cared for in the UH may have come from farther away. Moreover, patients cared for in PH or CH almost never lived nearer to the UH than the center where they were cared for. The map of patients’ geographical distribution around the center where they are cared for is presented in supplemental file in the Fig. 2 which illustrates patients’ preferences for proximity centers and the capability of the UH to recruit both patients living near or far from the center.

**Table 1:** Patients’ characteristics and treatment adherence according to the care centers. *N* = 294

		Total		PH		CH		UH	
		*n*	%	*n*	%	*n*	%	*n*	%
Patients’ characteristics									
Gender	Male	153	52	51	62	34	49	68	48
	Female	141	48	31	38	36	51	74	52
Age (tercile)	t1 (until 59y)	88	30	24	29	20	29	44	31
	t2 (60y to 72y)	156	53	40	49	37	53	79	56
	t3 (73y and more)	50	17	18	22	13	19	19	13
Comorbidity	None	117	40	36	44	26	37	55	39
	At least 1	177	60	46	56	44	63	87	61
Performance status (0 / 1–4)	Normal	234	80	67	82	45	64	122	86
	Degraded	60	20	15	18	25	36	20	14
Social deprivation index	Highly fav.	63	21	18	22	10	14	35	25
	Fav.	43	15	14	17	13	19	16	11
	Intermediate	68	23	22	27	18	26	28	20
	Dep.	74	25	13	16	19	27	42	30
	Highly dep.	46	16	15	18	10	14	21	15
Nearest care center	UH	119	40	8	10	0	0	111	78
	Other	175	60	74	90	70	100	31	22
Distance (km) to the care center	Mean (SD)	38	(59)	23	(30)	28	(29)	51	(76)
	Med [q1-q3]	20	[7; 47]	12	[4;30]	24	[7; 39]	23	[10; 68]
Distance (km) to the teaching hospital	Mean (SD)	87	(74)	123	(62)	119	(42)	51	(76)
	Med [q1-q3]	75	[22; 137]	128	[69;176]	113	[81;155]	23	[10; 71]
Disease characteristics									
Stage	I/II	123	*42*	44	54	26	*37*	53	37
	III/IV	171	*58*	38	*46*	44	*63*	89	63
LDH: lactate dehygrogenase	Normal	163	*55*	50	*61*	49	*70*	64	45
	Elevated	131	*45*	32	*39*	21	*30*	78	55
B symptoms	Absence	255	*87*	75	*91*	55	*79*	125	88
	Presence	39	*13*	7	9	15	*21*	17	12
Cancer management									
Hospitalization for toxicity	None	240	*82*	71	87	44	*63*	125	88
	At least 1 day	54	*18*	11	13	26	*37*	17	12
Relative dose intensity (RDI)	Mean (SD)	0.97	(0.10)	0.95	(0.11)	0.95	(0.12)	0.98	(0.08)
	Med [q1-q3]	1	[1; 1]	1	[0.9; 1]	1	[0.9; 1]	1	[1; 1]
Relative Dose Intensity (RDI)	RDI = 100 %	229	*78*	58	71	50	*71*	121	*85*
	RDI < 100 %	65	22	24	29	20	*29*	21	*15*

Table 2 presents the factors associated with being cared for somewhere else than the UH. Compared with patients in the UH, those in CH were more likely to have degraded performance status, and better initial prognosis. These patients also tended to live in a less favorable socioeconomic area, more specifically they tended to live more often in favored (*p* = 0.033) areas than in highly favored areas. Compared with patients in the UH, those in PH were more likely to have earlier stage tumor, and to live less than 40 km from their care center. These patients also tended to have degraded performance status but this was not statistically significant with an alpha risk fixed at 5 % (*p*-value = 0.072),

**Table 2:** Factors associated with being cared for somewhere other than in the Toulouse public funding University Hospital (UH) among patients treated by standard regimens (RCHOP or RCHVP) (*n* = 294)

		Being cared for the public funding community hospitals (CH) instead of in UH				Being cared for in the private funding for-profit hospitals (PH) instead of in UH			
		Model 1a				Model 1b			
		OR [95 % CI] *p-value*				OR [95 % CI] *p-value*			
Age (tertile)	t1 (until 59y)	1				1			
	t2 (60y to 72y)	0.9	[0.4;	1.9]	0.777	0.9	[0.4;	1.8]	0.749
	t3 (73y and more)	1.3	[0.5;	3.6]	0.586	2.1	[0.8;	5.2]	0.127
Gender	Men	1				1			
	Women	0.9	[0.5;	1.8]	0.818	0.6	[0.4;	1.2]	0.152
Comorbidity	None	1				1			
	At least 1	1.2	[0.6;	2.3]	0.676	0.9	[0.5;	1.7]	0.795
Performance status	Normal	1				1			
	Degraded	4.3	[1.9;	9.5]	0.000	2.2	[0.9;	5.1]	0.072
Distance to the care center (km)	Up to 13 km	1				1			
	14 – 41 km	1.5	[0.7;	3.3]	0.296	0.5	[0.2;	1.1]	0.070
	At least 42 km	0.5	[0.2;	1.3]	0.158	0.2	[0.1;	0.5]	0.000
Social deprivation index	Highly favored	1				1			
	Favored	3.2	[1.1;	9.4]	0.033	2.0	[0.7;	5.2]	0.182
	Intermediate	2.7	[1.0;	7.6]	0.054	1.8	[0.7;	4.5]	0.184
	Deprived	2.5	[0.9;	6.7]	0.073	0.7	[0.3;	1.9]	0.519
	Highly deprived	2.4	[0.7;	7.5]	0.143	1.6	[0.6;	4.1]	0.370
Stage	I/II	1				1			
	III/IV	0.9	[0.4;	1.7]	0.645	0.5	[0.3;	0.9]	0.026
B symptoms	Absence	1				1			
	Presence	1.6	[0.7;	4.0]	0.271	0.6	[0.2;	1.7]	0.356
Lactate dehydrogenase	Normal	1				1			
	Elevated	0.3	[0.1;	0.6]	0.000	0.6	[0.3;	1.2]	0.145

Global *p*-values in model 1a age: *p* = 0.704, social deprivation index: *p* = 0.226, distance to the care center: *p* < 0.055; in model 1b age: *p* = 0.135, social deprivation index: *p* = 0.186, distance to the care center: *p* = 0.001

Table 3 shows that being treated using the standard R-CHOP or R-CHVP protocols in UH rather than elsewhere was associated with lower rate of low RDI. After accounting successively for patients’ biomedical characteristics, their socio-economical characteristics and distance to care center, low RDI was more frequent in PH than in UH (model 2c, OR [95 % CI] = 3.3 [1.5; 7.1]) and CH (model 2c, OR [95 % CI] = 2.6 [1.2; 5.6]). The adjustment for hospitalization for toxicities altered the OR associated with CH which became no more statistically significant (model 2d, OR [95 % CI] = 1.9 [0.8; 4.4]). We have tested the center effect using a random intercept which was not presented as its variance was smaller than 10^−8^ after adjustment for the type of care provider. This may translate the fact the center-related variations in the probability of low RDI was completely explained by the type of the care center. In addition, the models showed that an advanced stage at diagnosis and at least one day of hospitalization for toxicity were more likely to result in low RDI. No effects were noted for age, comorbidity, initial prognosis, social deprivation level, and distance to center. Similar results were obtained in sensitivity analyzes using RDI as a continuous variable (data not shown).

**Table 3:** Factors associated with having a low RDI (RDI < 100 %) among DLBCL patients treated with CHOP21/RCHVP (*n* = 294)

		Model 2a				Model 2b				Model 2c				Model 2d			
		beta [95 % CI] *p-value*				beta [95 % CI] *p-value*				beta [95 % CI] *p-value*				beta [95 % CI] *p-value*			
Age	56 – 65 y					1				1				1			
	66 to 75y					0.4	[0.2;	0.8]	0.008	0.4	[0.2;	0.8]	0.014	0.4	[0.2;	0.8]	0.010
	At least 76y					0.6	[0.2;	1.4]	0.206	0.6	[0.3;	1.5]	0.265	0.6	[0.2;	1.4]	0.246
Gender	Men					1				1				1			
	Women					1.4	[0.8;	2.6]	0.242	1.4	[0.8;	2.6]	0.233	1.4	[0.8;	2.6]	0.251
Comorbidity	None					1				1				1			
	At least 1					1.2	[0.6;	2.2]	0.572	1.2	[0.6;	2.2]	0.653	1.2	[0.6;	2.3]	0.583
Performance status	Normal					1				1				1			
	Degraded					1.5	[0.7;	3.1]	0.310	1.4	[0.7;	3.1]	0.354	1.3	[0.6;	2.9]	0.480
Stage	I/II					1				1				1			
	III/IV					1.4	[0.8;	2.7]	0.282	1.5	[0.8;	2.8]	0.211	1.5	[0.8;	2.8]	0.235
B symptoms	Absence					1				1				1			
	Presence					1.4	[0.6;	3.1]	0.472	1.4	[0.6;	3.2]	0.441	1.2	[0.5;	2.9]	0.626
Lactate dehydrogenase	Normal					1				1				1			
	Elevated					1.2	[0.7;	2.3]	0.491	1.1	[0.6;	2.1]	0.679	1.1	[0.6;	2.0]	0.814
Social deprivation index	Highly fav.									1				1			
	Favored									0.6	[0.2;	1.8]	0.403	0.7	[0.2;	2.0]	0.508
	Intermediate									0.7	[0.3;	1.7]	0.387	0.7	[0.3;	1.8]	0.444
	Deprived									1.1	[0.5;	2.7]	0.842	1.1	[0.5;	2.8]	0.815
	Highly dep.									1.0	[0.4;	2.6]	0.993	1.0	[0.4;	2.6]	0.935
Distance to the care center (km)	Up to 13 km									1				1			
	14 – 40 km									1.0	[0.5;	2.1]	0.995	1.2	[0.6;	2.5]	0.687
	At least 41 km									1.6	[0.7;	3.3]	0.266	1.5	[0.7;	3.3]	0.323
Hospitalization for toxicity	None													1			
	At least 1 day													2.8	[1.4;	5.7]	0.006
Type of care center	UH	1				1				1				1			
	CH	2.3	[1.2;	4.6]	0.019	2.2	[1.0;	4.8]	0.039	2.6	[1.2;	5.6]	0.020	1.9	[0.8;	4.4]	0.120
	PH	2.4	[1.2;	4.6]	0.010	2.8	[1.4;	5.6]	0.005	3.3	[1.6;	7.1]	0.002	3.3	[1.5;	7.1]	0.002

All the models were adjusted for centre effect using a random intercept which is not presented as its variance in all the models was smaller than 10^−8^, this may be interpreted as a random intercept variance not statistically different from zero. Global *p*-values in model 2a type of care center: *p* < 0.016; in model 2b age: *p* = 0.029, type of care center: *p* = 0.013; in model 2c age: *p* = 0.050, social deprivation index: *p* = 0.709, distance to the care center: *p* = 0.456, type of care center: *p* = 0.005; in model 2d age: *p* = 0.037, social deprivation index: *p* = 0.805, distance to the care center: *p* = 0.612, type of care center: *p* = 0.009

## Discussion

This study, based on an ongoing cohort of DLBCL patients treated in the French Midi-Pyrénées region, shows an independent beneficial effect on RDI in the UH. PH, CH and UH cared for populations with different biomedical and socio-spatial characteristics. The former centers were more likely to treat patients living in their vicinity roughly within a 40 km radius. Patients treated in UH had more advanced stage and poorer initial prognosis without limitation regarding the distance from the residence to the care center. Patients’ recruitment profile across the different types of care providers failed to explain the difference in RDI between UH and PH or CH. In this study among patients only treated with standard protocols, low RDI was less often observed in UH than elsewhere. This may translate a potentially greater quality in cancer care and management specific to this center, despite the universal healthcare system setting.

One of the main limitations of this study is that we cannot generalize our results to the national level and to other French teaching hospitals in general. However, this study dealt with the fourth more densely populated regions in France according to the French National Institute of Statistic and Economics Studies (INSEE) [32] and the largest region of mainland France in terms of size. In addition, as is often observed in the rest of the country, regional healthcare is heterogeneously distributed around large urban area wherein multidisciplinary team meetings held in the UH centralize decisions regarding the management of both in- and outpatients, especially those needing intense treatment. At last, we excluded 42 patients from our analyzes because of missing data, decease or tumor progression during the treatment. These patients were evenly spread through the three center types. The comparison of the characteristics of included and excluded patients suggested that our sample overrepresented younger patients with less advanced stage tumor, better initial prognosis, less often hospitalization for toxicity and more often high RDI. The patients we studied would thus have a more favorable profile than those usually encountered in the current practice. We assume that caring patients with less favorable profile necessitate more discussion within the medical team and consequently it would be more affected by center-related disparity in cancer management.

This study has also several strengths. Few studies have explored the role of care provider types on treatment adherence in common practice in a universal healthcare system setting. We used a large (*n* = 294) DLBCL population homogeneously treated with the standard R-CHOP or R-CHVP protocols. Moreover, we collected data on biomedical and socio-spatial characteristics of patients, including the distance from residence to the reference center and to the care center where patients went, and treatments; this allowed us to study the effect of the types of care center among patients receiving the a same standard treatment.

Studies in solid tumors have already documented the influence of the care center characteristics on cancer care and patients outcomes. [33] Some evidence mainly in the United States and Northern European countries supports the benefit of being cared for in high-volume or academic hospitals, such as improved survival in lung [34] and colorectal cancers, [35] and improved survival and adherence to the guidelines in ovarian [36] and breast cancers. [37, 38] Regarding hematological malignancies, results from a population-based study in Nebraska showed a benefit of being cared for in urban university-based centers on survival among high risk lymphoma patients. [15] In more recent studies, the type of care center was also linked to overall survival showing a benefit of being treated in high volume hospital among acute myeloid leukemia [39] and DLBCL. [16] However, the underlying mechanism remains unclear. In the present study, we showed that higher RDI observed in the UH was not due to patient’s characteristics or treatment. This may suggest differential quality of care according to the center. The real stake is to identify what characteristics of the center may contribute to higher RDI in order to disseminate to the other centers and to ensure to all patients, whatever the center where are cared for, the best possible management of their cancer. Indeed, an organizational intervention has been implemented since 2006 among DLBCL patients treated in the UH of Midi-Pyrénées: an oncology-certified nurse systematically calls the patients during the treatment phase and collects clinical and biological observations, which are forwarded to the oncologist to enhance the care management and adherence to the treatment plan. [40] Presently, the efficacy in reducing low RDI and the medico-economic evaluation of this nurse navigator procedure will be formally tested in a non-randomized-controlled study among several French teaching hospitals. We assume that such intervention may participate substantially to the mechanisms behind the higher RDI observed in the UH. In other clinical settings, studies have already given evidences of the benefits of nurse navigator interventions on cancer patients care management. [41–44] A possible solution may be to ensure such service for all patients across the region. However, other explanations are conceivable. For the past several years, studies have supported the existence of a noncompliance issue in medical oncology, pointing out the influence of physicians and care providers on adherence to the treatment plan was better in academic settings for both conventional [45] and targeted chemotherapy. [46] However, how the types of care providers may influence physician non-adherent behavior remains unclear. With regard to our study, we can rule out an effect of any lack of knowledge and weakness in clinician training as, regardless of the center where they practice, most of the clinicians have been trained by the Hematology Department of the UH. In addition, they regularly attend regional education sessions and national meetings which take place at least yearly. Another explanation might reside in the fact that reduction in treatment intensity outside academic settings may reflect more cautious care behavior among clinicians. Indeed, compared to clinicians in teaching hospitals, they may more frequently face frailer patients and limited facilities such as emergency or imaging units available 7 days a week and 24 h a day and therefore be inclined to reduce dose-intensity. Conversely, in the UH, the availability of facilities might encourage clinicians to not reduce dose-intensity which would be associated, at least in part, with an increase of hospitalization for toxicities. However, our results showed higher RDI in UH than PH even after controlling for hospitalization for toxicities. Moreover, hospitalizations for toxicities were more frequent in CH than elsewhere and adjustment for this variable altered the OR associated with CH which did not correspond to this explanation. In addition, the presence of the nurse navigator program in the UH may also encourage clinician to not reduce dose-intensity tendencies because it may provide a support to manage symptoms. This does not mean that the UH effect we observed is only the manifestation of the nurse navigator intervention, and we have to question whether such intervention would have been implemented elsewhere than in UH and with what outcome. Indeed, the “UH effect” on RDI we observed in this study may correspond to the expression of an enabling environment for developing innovative interventions. But whatever the reason, our results show differences between care centers that may be the consequence of different care management as potentially illustrated in UH. This point is major for us because it asks the question of how to insure the same quality of care everywhere on the French territory, which is an aim of the French health system.

## Conclusion

This observational study in French common clinical practice shows that even in a universal healthcare system, disparities do exist in the management of DLCBL patients treated by standard protocols according to the types of care center. Although the fact that the treatment plan of all patients has been initially discussed in MTM which involves doctors among who most were trained at the same facility, we found management discrepancies related to the type of the care facility. Indeed, we found a higher adhesion to the treatment plan associated with being treated in the regional teaching hospital, not due to differences in recruited patients or in treatment provided. This suggests that at least in our study population, attempts at standardizing care using a medical outreach team, practice guidelines and clinician performance assessments might be not sufficient to ensure equal access to high-quality care. In addition, the place that teaching hospitals should occupy in the care landscape appears to be a critical issue. The dissemination of innovative organizational interventions such as for instance the nurse navigator developed for DLBCL patients in the Toulouse UH to non-academic centers might improve quality of care.

## Abbreviations

CH, public funding community hospital; DLBCL, diffuse large B cells lymphoma; ECOG, Eastern Cooperative Oncology Group; HIV, human immunodeficiency virus; HL, Hodgkin Lymphoma; IPI: international prognostic index; MTM, multidisciplinary team meeting; NCCN, the national comprehensive cancer network of the United States of America; NHL, non-Hodgkin Lymphoma; PH, private funding for-profit hospital; PS, ECOG performance status; R-CHOP, rituximab, cyclophosphamide, doxorubicin, vincristine, prednisone; R-CHVP,rituximab, cyclophosphamide, doxorubicin, etoposide, prednisone; RDI, relative dose intensity; SES, socio-economical status; UH, public funding teaching hospital

## References

[1] Frederiksen BL, Brown PN, Dalton SO, Steding-Jessen M, Osler M (2011). Socioeconomic inequalities in prognostic markers of non-Hodgkin lymphoma: Analysis of a national clinical database. Eur J Cancer.

[2] Keegan TM, Clarke C, Chang E, Shema S, Glaser S (2009). Disparities in survival after Hodgkin lymphoma: a population-based study. Cancer Causes Control.

[3] Bray C, Morrison DS, McKay P (2008). Socio-economic deprivation and survival of non-Hodgkin lymphoma in Scotland. Leuk Lymphoma.

[4] Frederiksen BL, Dalton SO, Osler M, Steding-Jessen M, de Nully BP (2012). Socioeconomic position, treatment, and survival of non-Hodgkin lymphoma in Denmark - a nationwide study. Br J Cancer.

[5] Keegan THM, McClure LA, Foran JM, Clarke CA (2009). Improvements in Survival After Follicular Lymphoma by Race/Ethnicity and Socioeconomic Status: A Population-Based Study. J Clin Oncol.

[6] Simard JF, Baecklund F, Chang ET, Baecklund E, Hjalgrim H, Olov Adami H, Glimelius B, Smedby KE (2013). Lifestyle factors, autoimmune disease and family history in prognosis of non-hodgkin lymphoma overall and subtypes. Int J Cancer.

[7] Wang M, Burau KD, Fang S, Wang H, Du XL (2008). Ethnic variations in diagnosis, treatment, socioeconomic status, and survival in a large population-based cohort of elderly patients with non-Hodgkin lymphoma. Cancer.

[8] Kato I, Booza J, Quarshie WO, Schwartz K (2012). Persistent socioeconomic inequalities in cancer survival in the United States: 1973–2007 surveillance, epidemiology and end results (SEER) data for breast cancer and non-Hodgkin's lymphoma. J Registry Manag.

[9] Kristinsson SY, Derolf ÅR, Edgren G, Dickman PW, Björkholm M (2009). Socioeconomic Differences in Patient Survival Are Increasing for Acute Myeloid Leukemia and Multiple Myeloma in Sweden. J Clin Oncol.

[10] Lee B, Goktepe O, Hay K, Connors JM, Sehn LH, Savage KJ, Shenkier T, Klasa R, Gerrie A, Villa D (2014). Effect of Place of Residence and Treatment on Survival Outcomes in Patients With Diffuse Large B-Cell Lymphoma in British Columbia. Oncologist.

[11] Olszewski AJ, Shrestha R, Castillo JJ (2015). Treatment selection and outcomes in early-stage classical hodgkin lymphoma: analysis of the national cancer data base. J Clin Oncol.

[12] Keegan THM, Moy LM, Foran JM, Alizadeh AA, Chang ET, Shema SJ, Schupp CW, Clarke CA, Glaser SL (2013). Rituximab use and survival after diffuse large B-cell or follicular lymphoma: a population-based study. Leuk Lymphoma.

[13] Tao L, Foran JM, Clarke CA, Gomez SL, Keegan THM (2014). Socioeconomic disparities in mortality after diffuse large B-cell lymphoma in the modern treatment era. Blood.

[14] Paulson K, Lambert P, Bredeson C, Demers A, Nowatzki J, Richardson E, Rubinger M, Szwajcer D, Seftel MD (2010). Does location matter[quest] Rural vs urban outcomes after blood and marrow transplantation in a population-based Canadian cohort. Bone Marrow Transplant.

[15] Loberiza FR, Cannon AJ, Weisenburger DD, Vose JM, Moehr MJ, Bast MA, Bierman PJ, Bociek RG, Armitage JO (2009). Survival disparities in patients with lymphoma according to place of residence and treatment provider: a population-based study. J Clin Oncol.

[16] Borel C, Lamy S, Compaci G, Récher C, Jeannot P, Nogarot JC, Bauvin E, Despas F, Delpierre C, Laurent G. Non-medical determinants of adherence to R-CHOP therapy for diffuse large B-cell lymphoma: implication for survival. BMC cancer. 2014;15(1):1–11. 10.1186/s12885-015-1287-9 PMC4403884 25884669

[17] Hirakawa T, Yamaguchi H, Yokose N, Gomi S, Inokuchi K, Dan K (2010). Importance of maintaining the relative dose intensity of CHOP-like regimens combined with rituximab in patients with diffuse large B-cell lymphoma. Ann Hematol.

[18] Terada Y, Nakamae H, Aimoto R, Kanashima H, Sakamoto E, Aimoto M, Inoue E, Koh H, Nakane T, Takeoka Y (2009). Impact of relative dose intensity (RDI) in CHOP combined with rituximab (R-CHOP) on survival in diffuse large B-cell lymphoma. J Exp Clin Cancer Res.

[19] Bouche G, Migeot V, Mathoulin-Pélissier S, Salamon R, Ingrand P (2008). Breast cancer surgery: Do all patients want to go to high-volume hospitals?. Surgery.

[20] Pfreundschuh M, Trümper L, Österborg A, Pettengell R, Trneny M, Imrie K, Ma D, Gill D, Walewski J, Zinzani P-L (2006). CHOP-like chemotherapy plus rituximab versus CHOP-like chemotherapy alone in young patients with good-prognosis diffuse large-B-cell lymphoma: a randomised controlled trial by the MabThera International Trial (MInT) Group. Lancet Oncol.

[21] Coiffier B, Lepage E, Brière J, Herbrecht R, Tilly H, Bouabdallah R, Morel P, Van Den Neste E, Salles G, Gaulard P (2002). CHOP Chemotherapy plus Rituximab Compared with CHOP Alone in Elderly Patients with Diffuse Large-B-Cell Lymphoma. N Engl J Med.

[22] Oken MM, Creech RH, Tormey DC, Horton J, Davis TE, McFadden ET, Carbone PP (1982). Toxicity and response criteria of the Eastern cooperative oncology group. Am J Clin Oncol.

[23] Blay JY, Gomez F, Sebban C, Bachelot T, Biron P, Guglielmi C, Hagenbeek A, Somers R, Chauvin F, Philip T (1998). The international prognostic index correlates to survival in patients with aggressive lymphoma in relapse: analysis of the PARMA trial. Blood.

[24] The International Non-Hodgkin's Lymphoma Prognostic Factors Project (1993). A predictive model for aggressive non-hodgkin’s lymphoma. N Engl J Med.

[25] Zhou Z, Sehn LH, Rademaker AW, Gordon LI, LaCasce AS, Crosby-Thompson A, Vanderplas A, Zelenetz AD, Abel GA, Rodriguez MA (2014). An enhanced international prognostic index (NCCN-IPI) for patients with diffuse large B-cell lymphoma treated in the rituximab era. Blood.

[26] Fitoussi O, Belhadj K, Mounier N, Parrens M, Tilly H, Salles G, Feugier P, Ferme C, Ysebaert L, Gabarre J (2011). Survival impact of rituximab combined with ACVBP and upfront consolidation autotransplantation in high-risk diffuse large B-cell lymphoma for GELA. Haematologica.

[27] Peyrade F, Jardin F, Thieblemont C, Thyss A, Emile J-F, Castaigne S, Coiffier B, Haioun C, Bologna S, Fitoussi O (2011). Attenuated immunochemotherapy regimen (R-miniCHOP) in elderly patients older than 80 years with diffuse large B-cell lymphoma: a multicentre, single-arm, phase 2 trial. Lancet Oncol.

[28] Pfreundschuh M, Schubert J, Ziepert M, Schmits R, Mohren M, Lengfelder E, Reiser M, Nickenig C, Clemens M, Peter N (2008). Six versus eight cycles of bi-weekly CHOP-14 with or without rituximab in elderly patients with aggressive CD20+ B-cell lymphomas: a randomised controlled trial (RICOVER-60). Lancet Oncol.

[29] Pornet C, Delpierre C, Dejardin O, Grosclaude P, Launay L, Guittet L, Lang T, Launoy G (2012). Construction of an adaptable European transnational ecological deprivation index: the French version. J Epidemiol Community Health.

[30] Epelbaum R, Faraggi D, Ben-Arie Y, Ben-Shahar M, Haim N, Ron Y, Robinson E, Cohen Y (1990). Survival of diffuse large cell lymphoma. A multivariate analysis including dose intensity variables. Cancer.

[31] R Development Core Team (2008). R: A language and environment for statistical computing.

[32] Size of most densely populated French cities (last update) [http://www.insee.fr/fr/themes/tableau.asp?reg_id=0&ref_id=nattef01214]

[33] Hillner BE, Smith TJ, Desch CE (2000). Hospital and physician volume or specialization and outcomes in cancer treatment: importance in quality of cancer care. J Clin Oncol.

[34] Cheung M, Hamilton K, Sherman R, Byrne M, Nguyen D, Franceschi D, Koniaris L (2009). Impact of teaching facility status and high-volume centers on outcomes for lung cancer resection: an examination of 13,469 surgical patients. Ann Surg Oncol.

[35] Etzioni DA, Young-Fadok TM, Cima RR, Wasif N, Madoff RD, Naessens JM, Habermann EB (2014). Patient survival after surgical treatment of rectal cancer: Impact of surgeon and hospital characteristics. Cancer.

[36] Cliby WA, Powell MA, Al-Hammadi N, Chen L, Philip Miller J, Roland PY, Mutch DG, Bristow RE (2014). Ovarian cancer in the United States: contemporary patterns of care associated with improved survival. Gynecol Oncol.

[37] Vrijens F, Stordeur S, Beirens K, Devriese S, Van Eycken E, Vlayen J (2012). Effect of hospital volume on processes of care and 5-year survival after breast cancer: A population-based study on 25 000 women. Breast.

[38] Siesling S, Tjan-Heijnen VG, de Roos M, Snel Y, van Dalen T, Wouters M, Struikmans H, van der Hoeven JM, Maduro J, Visser O (2014). Impact of hospital volume on breast cancer outcome: a population-based study in the Netherlands. Breast Cancer Res Treat.

[39] Giri S, Pathak R, Aryal MR, Karmacharya P, Bhatt VR, Martin MG (2015). Impact of hospital volume on outcomes of patients undergoing chemotherapy for acute myeloid leukemia: a matched cohort study. Blood.

[40] Compaci G, Ysebaert L, Obéric L, Derumeaux H, Laurent G (2011). Effectiveness of telephone support during chemotherapy in patients with diffuse large B cell lymphoma: the ambulatory medical assistance (AMA) experience. Int J Nurs Stud.

[41] Wagner EH, Ludman EJ, Aiello Bowles EJ, Penfold R, Reid RJ, Rutter CM, Chubak J, McCorkle R (2014). Nurse navigators in early cancer care: a randomized. Control Trial J Clin Oncol.

[42] Freeman HP, Muth BJ, Kerner JF (1995). Expanding access to cancer screening and clinical follow-up among the medically underserved. Cancer Pract.

[43] Lee T, Ko I, Lee I, Kim E, Shin M, Roh S, Yoon D, Choi S, Chang H (2011). Effects of nurse navigators on health outcomes of cancer patients. Cancer Nurs.

[44] McMullen L (2013). Oncology nurse navigators and the continuum of cancer care. Semin Oncol Nurs.

[45] Schleifer SJ, Bhardwaj S, Lebovits A, Tanaka JS, Messe M, Strain JJ (1991). Predictors of physician nonadherence to chemotherapy regimens. Cancer.

[46] Noens L, van Lierde MA, De Bock R, Verhoef G, Zachee P, Berneman Z, Martiat P, Mineur P, Van Eygen K, MacDonald K (2009). Prevalence, determinants, and outcomes of nonadherence to imatinib therapy in patients with chronic myeloid leukemia: the ADAGIO study. Blood.

